# Phytochrome and Ethylene Signaling Integration in *Arabidopsis* Occurs via the Transcriptional Regulation of Genes Co-targeted by PIFs and EIN3

**DOI:** 10.3389/fpls.2016.01055

**Published:** 2016-07-19

**Authors:** Jinkil Jeong, Keunhwa Kim, Mi E. Kim, Hye G. Kim, Gwi S. Heo, Ohkmae K. Park, Youn-Il Park, Giltsu Choi, Eunkyoo Oh

**Affiliations:** ^1^Department of Biological Sciences, Korea Advanced Institute of Science and TechnologyDaejeon, South Korea; ^2^Center for Gas Analysis, Korea Research Institute of Standards and ScienceDaejeon, South Korea; ^3^School of Life Sciences and Biotechnology, Korea UniversitySeoul, South Korea; ^4^Department of Bioscience and Biotechnology, Chungnam National UniversityDaejeon, South Korea; ^5^Department of Bioenergy Science and Technology, Chonnam National UniversityGwangju, South Korea

**Keywords:** phytochrome, ethylene signaling, phytochrome-interacting factors PIFs, EIN3, transcription factors, signaling crosstalk, Photobleaching

## Abstract

Plant seedlings germinating under the soil are challenged by rough soil grains that can induce physical damage and sudden exposure to light, which can induce photobleaching. Seedlings overcome these challenges by developing apical hooks and by suppressing chlorophyll precursor biosynthesis. These adaptive responses are, respectively, regulated by the phytochrome and ethylene signaling pathways via the PHYTOCHROME-INTERACTING FACTORs (PIFs) and the ETHYLENE INSENSITIVE 3 (EIN3)/EIN3-LIKE transcription factors. Although many processes downstream of phytochrome and ethylene signaling are similar, it remains unclear if and where these pathways converge. Here, we show PIFs and EIN3 induce similar changes in the transcriptome without robustly regulating each other’s signaling pathways. PIFs and EIN3 target highly overlapped gene promoters and activate subsets of the co-target genes either interdependently or additively to induce plant responses. For chlorophyll biosynthesis, PIFs and EIN3 target and interdependently activate the expression of *HOOKLESS1*. HOOKLESS1, in turn, represses chlorophyll synthesis genes to prevent photobleaching. Thus, our results indicate an integration of the phytochrome and ethylene signaling pathways at the level of transcriptional gene regulation by two core groups of transcription factors, PIFs and EIN3.

## Introduction

Plants use light not only as an energy source but also as a signal that allows them to monitor their environment and neighboring plants. Plants have multiple types of photoreceptors including phytochromes, cryptochromes, phototropins, zeitlupes, and UVR8. This photoreceptor diversity allows plants to detect a broad spectrum of light stimuli and respond with a wide range of developmental and physiological processes. The phytochromes perceive red and far-red light and respond by regulating seed germination, photomorphogenesis, shade avoidance, and senescence (Mathews, 2006; Franklin and Quail, 2010). In the dark, cytosolic phytochromes exist in an inactive Pr form. Light exposure induces a conformational change to the active Pfr form, which then translocates to the nucleus (Sakamoto and Nagatani, 1996; Kircher et al., 1999; Yamaguchi et al., 1999). In the nucleus, active phytochromes interact with various phytochrome-interacting factors to trigger the global gene expression changes that direct appropriate light responses (Castillon et al., 2007; Bae and Choi, 2008; Leivar and Quail, 2011).

The PIFs (i.e., PIF1, PIF3, PIF4, PIF5, and PIF7) are a group of well-characterized bHLH transcription factors that preferentially interact with active Pfr phytochrome (Ni et al., 1998; Huq and Quail, 2002; Huq et al., 2004; Khanna et al., 2004; Oh et al., 2004). This interaction inhibits the PIFs, either by dissociating them from their target promoters or by inducing their phosphorylation and subsequent degradation by the 26S proteasome (Bauer et al., 2004; Park et al., 2004; Shen et al., 2005; Al-Sady et al., 2006; Lorrain et al., 2008). PIFs inhibit phytochrome-mediated light responses like seed germination (PIF1), seedling photomorphogenesis (PIF1, PIF3, PIF4, and PIF5), shade avoidance (PIF4, PIF5, and PIF7), and senescence (PIF4 and PIF5). The *pif* quadruple mutant (*pif1*/*pif3*/*pif4*/*pif5*, *pifq*) shows constitutive photomorphogenic phenotypes in the dark including short hypocotyls, opened cotyledons without apical hooks, and hypocotyl agravitropism (Leivar et al., 2008b; Shin et al., 2009). In addition, etiolated *pifq* seedlings accumulate the precursor of chlorophyll protochlorophyllide, which causes photo-oxidation and bleaching upon sudden light exposure (Huq et al., 2004; Shin et al., 2009; Stephenson et al., 2009). In etiolated wild type seedlings, PIF1 and PIF3 inhibit several chlorophyll-biosynthesis genes [e.g., *HEMA1* and *CHLH*/*GENOME UNCOUPLED5* (*CHELATASE H*/*GUN5*)] to reduce the accumulation of protochlorophyllide and activate the protochlorophyllide oxidoreductases (POR) that convert protochlorophyllide to chlorophylls upon light exposure (Moon et al., 2008; Shin et al., 2009; Stephenson et al., 2009). Thus, PIFs prevent seedling photobleaching by inhibiting the over-accumulation of free protochlorophyllide during emergence.

Ethylene, a gaseous plant hormone, induces in etiolated seedlings the so-called “triple response” of a short, thickened hypocotyl and a root with an exaggerated apical hook. Ethylene is synthesized from methionine through S-adenosylmethionine (SAM) and 1-aminocyclopropane-1-carboxylic acid (ACC) intermediates (Adams and Yang, 1979). ACC synthase (ACS) catalyzes the conversion of SAM to ACC, which is the committed step in ethylene biosynthesis. Then, ACC oxidase (ACO) converts ACC to ethylene. In the absence of ethylene, ethylene receptors (e.g., ETHYELENE RESISTENT 1 (ETR1) and ETR2) act with CONSTITUTIVE TRIPLE RESPONSE 1 (CTR1) to inhibit ETHYLENE INSENSITIVE 2 (EIN2). This, in turn, inhibits the degradation of two transcription factors, ETHYLENE INSENSITIVE 3 (EIN3) and EIN3-LIKE 1 (EIL1) via EBF1 and EBF2, or inhibits the translation of EBF1 and 2 (Zhao and Guo, 2011; Merchante et al., 2013, 2015; Li et al., 2015). Ethylene binds and inhibits the ethylene receptors to stabilize EIN3 and EIL1. The stabilized EIN3 and EIL1 then regulate various downstream targets, including the *ETHYLENE RESPONSE FACTOR*s (*ERF*s) to induce ethylene responses (Chao et al., 1997; Solano et al., 1998). Another downstream signaling gene, *HOOKLESS1* (*HLS1*, an N-acetyltransferase), is important for ethylene-mediated apical hook formation (Lehman et al., 1996). In short, ethylene signaling is essential for the survival of emerging seedlings (Zhong et al., 2014).

Phytochrome-interacting factors regulate several developmental processes via crosstalk with hormone signaling pathways. For example, PIF1 inhibits seed germination in the dark in part by directly activating the expression of gibberellin (GA) and abscisic acid (ABA) signaling genes like *GIBBERELLIN INSENSITIVE* (*GA INSENSITIVE*, *GAI*), *REPRESSOR OF GA1* (*RGA1*), *ABSCISIC ACID INSENSITIVE3* (*ABA INSENSITIVE3*, *ABI3*), and *ABI5* (Oh et al., 2009). It also indirectly regulates GA and ABA metabolic genes to increase ABA levels and decrease GA levels (Oh et al., 2009). BZR1 and ARF6 are key transcription factors in brassinosteroid (BR) and auxin signaling, respectively. In seedlings, PIF4 directly binds BZR1 and ARF6 to cooperatively bind and regulate the promoters of many shared target genes (Oh et al., 2012, 2014). These shared targets include the *PACLOBUTRAZOL RESISTANCE* (*PRE*) family of factors that induce hypocotyl elongation in response to hormonal and environmental signals. GA increases the activities of the PIFs by destabilizing DELLA proteins like GAI and RGA. DELLA proteins directly bind and inhibit PIF DNA-binding and indirectly inhibit the formation of BZR1-PIF4 complexes on target promoters via their interaction with BZR1. BR increases PIF activity either by directly stabilizing PIF4 or by indirectly activating BZR1 and inducing the formation of BZR1-PIF4 complexes.

Several lines of evidence suggest significant crosstalk between phytochrome and ethylene signaling in *Arabidopsis* seedling development. First, *PIF5* overexpression increases ethylene levels in etiolated seedlings (Khanna et al., 2007) by directly binding and activating the *ACS* promoter (Gallego-Bartolome et al., 2011; Oh et al., 2012). Second, PIF1 and EIN3/EIL1 inhibit photobleaching by inhibiting the expression of protochlorophyllide biosynthetic genes and activating the expression of *POR* genes (Zhong et al., 2009). Although both PIF1 and EIN3/EIL1 inhibit photobleaching, they seem to function independently as exogenous ACC rescues the excessive photobleaching of *pif1* mutants. Third, ethylene promotes hypocotyl elongation in light-grown but not dark-grown seedlings by increasing *PIF3* expression (Zhong et al., 2012). Fourth, EIN3 and EIL1 inhibit photobleaching in dark-grown seedlings by directly up-regulating *PIF3* (Zhong et al., 2014). Contrary to results with *pif1* mutants, exogenous ACC treatment does not rescue the excessive bleaching of *pif3* mutants. This suggests ethylene inhibits photobleaching by increasing *PIF3* mRNA levels.

Although the PIFs each preferentially regulate specific responses, there is redundancy in their regulation of various aspects of seedling development including seedling morphology, photobleaching, and hypocotyl negative gravitropism. Thus, it is important to determine how PIFs in general interact with other signaling pathways in dark-grown seedlings. Here, we present a systematic study of the relationship between the PIFs and ethylene signaling. The global transcriptional profile of etiolated *pifq* mutant seedlings is itself suggestive of reduced ethylene signaling. This is consistent with the phenotypic similarities between *pifq* mutant seedlings and and ethylene-insensitive mutant seedlings. Unexpectedly, however, the lack of PIFs in *pifq* mutants neither suppresses ethylene biosynthesis during etiolation nor induces significant changes in EIN3 stability. Instead, PIFs and EIN3 bind a highly overlapping set of target genes without affecting one another’s DNA-binding ability. This binding activates their co-targeted genes either interdependently or additively. One of these co-targeted genes, *HLS1*, prevents photobleaching by regulating the expression of chlorophyll synthesis genes. Together, our results demonstrate the phytochrome and ethylene signaling pathways converge at the promoters of genes simultaneously targeted by PIFs and EIN3.

## Materials and Methods

### Plant Materials and Growth Conditions

*Arabidopsis thaliana* plants were grown at 22–24°C under long days (16 h light/8 h dark) in a growth room with cool-white fluorescent light (90–100 μmol m^-2^ s^-1^) for general growth and seed harvest. Mutants and transgenic lines are described in Supplementary Table S1. For phenotypic analyses, surface-sterilized seeds were plated on Murashige and Skoog (MS, Duchefa, M0222) agar plates (half-strength MS, 0.8% phytoagar, and 0.05% MES, pH 5.7), stratified for 3 days at 4°C in darkness, transferred to white light for 3 h to synchronize seed germination, and grown in different experimental conditions. ACC (Sigma, A3903) was dissolved in water, ethephon (Sigma, C-0143) was dissolved in DMSO, and AgNO_3_ (Sigma, S-0139) was dissolved in either water or DMSO depending on the experiment. For molecular experiments, seedlings were grown under darkness or under red light (13 μmol m^-2^ s^-1^) for the indicated period.

### Photobleaching and Protochlorophyllide Levels

For photobleaching assays, seedlings grown in the dark for the indicated period were transferred to continuous white light (100 μmol⋅m^-2^⋅s^-1^) for 3 days. Then, bleached seedlings were counted. For protochlorophyllide quantification, 10 seedlings grown in the dark for 4 days were gently agitated in 1 mL of ice-cold 80% acetone for 1 h in the dark at 4°C to extract pigments. The protochlorophyllide level in 100 μl of supernatant was determined using a fluorescence spectrophotometer (Tecan, infinite 200 PRO) with an excitation wavelength of 440 nm, a bandwidth of 4 nm, and an emission wavelength of 600–720 nm.

### Ethylene Levels

Ethylene levels were measured in 100 seedlings grown in 14 ml vials containing 10 ml growth medium and 4 ml headspace. The vials were refreshed with hydrocarbon-free air before they were sealed gas-tight and further incubated for 24 h in the dark. The headspace air was retrieved and ethylene was quantified by gas chromatography (Hewlett-Packard, 5890 series II).

### qPCR

Total RNA from plant tissues was isolated using a plant total RNA extraction kit (Sigma). First-strand cDNAs were prepared with 2 μg of total RNA and M-MLV reverse transcriptase (Promega) according to the manufacturer’s instructions. Gene expression levels were determined by qPCR using SYBR green on a CFX Connect machine (Bio-Rad). Gene expression was normalized to *PP2A* as an internal control. The gene-specific primers used for qPCR are listed in Supplementary Table S2.

### SDS-PAGE and Immunoblot Analysis

Seedlings were harvested and flash-frozen in liquid nitrogen under a dim green light. The seedlings were then ground in liquid nitrogen and homogenized in denaturing buffer (100 mM NaH_2_PO_4_, 10 mM Tris-HCl, 8 M urea, pH 8.0) by vigorous vortexing. The debris was removed by centrifugation at 20,000 ×*g* for 10 m at 4°C. For immunoblot analysis, the supernatants were separated on an 8% SDS-polyacrylamide gel. Then, the proteins were transferred to a nitrocellulose membrane (Hybond ECL, Amersham) using transfer buffer (5.8 g l^-1^ Tris base, 29 g l^-1^ glycine, 20% methanol, and 0.01% SDS). A rabbit polyclonal anti-EIN3 antibody for native EIN3 (Kim et al., 2013), a rabbit polyclonal anti-Myc antibody (Santa Cruz, CA, USA) for PIF4-Myc, a mouse monoclonal anti-FLAG antibody (Sigma, USA) for EIN3-FLAG, and a mouse monoclonal anti-tubulin antibody (Sigma, USA) for the loading control were used for protein detection. All antibodies were diluted in PBS buffer containing 0.05% Tween 20. Blots were washed three times with the same buffer and then incubated with the appropriate secondary antibodies. After washing three times, the horseradish peroxidase activity of the secondary antibodies was detected using an ECL detection kit (AbFRONTIER, Korea).

### Chromatin Immunoprecipitation (ChIP)

Plants overexpressing *GFP-Myc*, *PIF1-Myc*, *PIF3-Myc*, *PIF4-Myc*, *PIF5-Myc*, or *EIN3-FLAG* were grown for 4 days under the indicated conditions before cross-linking for 20 m with 1% formaldehyde under vacuum. Chromatin complexes were isolated and sonicated as described with slight modifications (Oh et al., 2009). An anti-Myc monoclonal antibody (mouse, Cell Signaling) or an anti-FLAG polyclonal antibody (rabbit, Sigma), and Protein A agarose/salmon sperm DNA (Millipore) were used for immunoprecipitation. After reverse cross-linking and protein digestion, DNA was purified using the QIAquick PCR Purification Kit (Qiagen) before being used for qPCR.

### Microarray and ChIP-Chip/Seq Analysis

All microarray analysis was performed with R version 2.15.0. The limma package was used for background correction and intra- and inter-array normalization. Then, lmFit was used to fit a linear model to the data so statistical calculations could be made using *ebayes*. ChIP-chip/Seq data was mapped to the TAIR10 genome using bowtie, analyzed by CisGenome v2.0, and visualized by IGV v2.3. Gene Set Enrichment Analysis was conducted with the GSEA package v2.08 (Broad Institute, MIT) according to the online user guide. ACC-responsive gene sets were generated based on published microarray data (Nemhauser et al., 2006).

### Accession Numbers

PIF1 (AT2G20180), PIF3 (AT1G09530), PIF4 (AT2G43010), PIF5 (AT3G59060), ETR1 (AT1G66340), ETR2 (AT3G23150), ERS1 (AT2G40940), ERS2 (AT1G04310), EIN4 (AT3G04580), CTR1 (AT5G03730), EIN2 (AT5G03280), EIN5 (AT1G54490), EBF2 (AT5G25350), EIN3 (AT3G20770), EIL1 (AT2G27050), EBP (AT3G16770), ACO1 (AT2G19590), ERF1 (AT3G23240), EXP9 (AT5G02260), FHL (AT5G02200), HB52 (AT5G53980), AHP1 (AT3G21510), HLS1 (AT4G37580), LOG5 (AT4G35190), GRF2 (AT4G37740), CEL1 (AT1G70710), BRG3 (AT3G12920), SBP1 (AT4G14030), GUN4 (AT3G59400), CHLH/GUN5 (AT5G13630), HEMA1 (AT1G58290), PORA (AT5G54190), PORB (AT4G27440), PORC (AT1G03630), PP2A (AT1G13320), EF1ALPHA (AT5G60390).

## Results

### The Transcriptome Profile of the *pif quadruple* Mutant Is Consistent with Reduced Ethylene Responses

**FIGURE 1:**
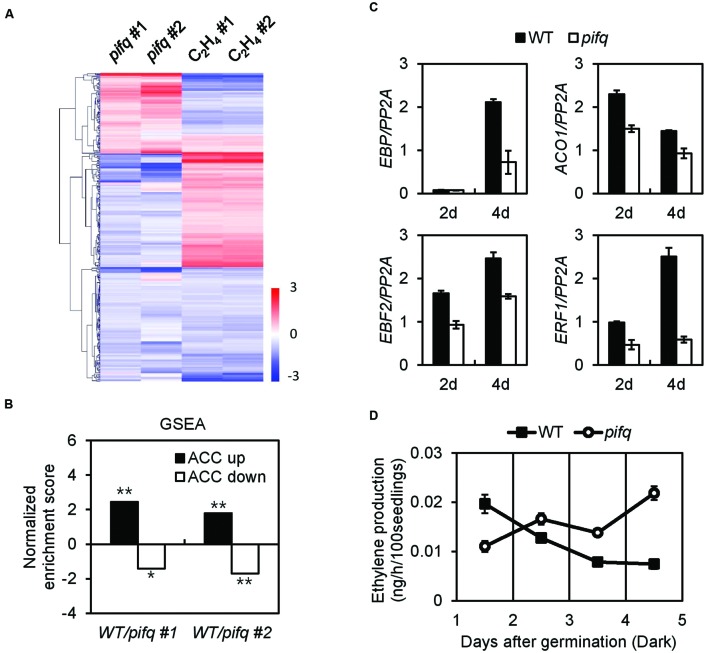
**Ethylene responses are suppressed in *pifq* mutants regardless of endogenous ethylene levels. (A)** Hierarchical clustering of two independent pifq microarrays and two independent ethylene treatment microarrays from dark-grown seedlings. 378 genes with *P* < 0.05 from the four microarrays were selected and analyzed. **(B)** Gene set enrichment analysis (GSEA) of the two independent *pifq* microarrays shows PIFs significantly up-regulate an ACC-inducible gene set and significantly down-regulate an ACC-repressible gene set (^∗^FDR = 0.086, ^∗∗^FDR < 0.005). **(C)** Expression of four representative ethylene marker genes from 2- and 4-day-old WT and *pifq* mutant etiolated seedlings. *PP2A* was used as an internal qPCR control. SD, *n* = 3 biological replicates. **(D)** Quantification of endogenous ethylene from WT and *pifq* mutants (SD, *n* = 3 biological replicates; ^∗^*P* < 0.05, Student’s *t*-test).

The *pifq* mutant and the ethylene-related mutants *etr1*, *ein2*, and *ein3* show open cotyledons in the dark and excessive photobleaching when transferred to the light (Supplementary Figure S1). This suggests PIF signaling and ethylene signaling interact. We therefore analyzed four published microarray datasets that compare dark-grown *pifq* mutants to wild type or ethylene-treated dark-grown wild type to non-treated wild type. These four microarray datasets overlap on 378 genes with significantly altered levels of expression (moderated t-statistic, *P* < 0.05). Of these 378 genes, 196 show an inverse correlation in the *pifq* mutants versus wild type ethylene-treated seedlings (**Figure 1A**). This suggests PIFs positively regulate many ethylene-responsive genes. We next used Gene Set Enrichment Analysis (GSEA) to determine whether ethylene-responsive genes are statistically enriched among PIF-regulated genes. For the analysis, we divided all ethylene-responsive genes into those showing up-regulation of the ethylene precursor ACC (ACC up) and those showing down-regulation of ACC (ACC down). We found ACC up-regulated genes tend to be significantly down-regulated in the *pifq* mutant, while ACC down-regulated genes tend to be significantly up-regulated in the *pifq* mutant (**Figure 1B**). We also observed suppression of several well-known ethylene-responsive markers in both 2- and 4-day-old dark-grown *pifq* seedlings (**Figure 1C**). Together, these results indicate PIFs and ethylene regulate large numbers of genes significantly in the same direction. This is consistent with the reduced ethylene signaling phenotypes of the *pifq* mutants.

### Ethylene-Responsive Gene Suppression in *pifq* Mutants Is Uncorrelated with Endogenous Ethylene Levels

**FIGURE 2:**
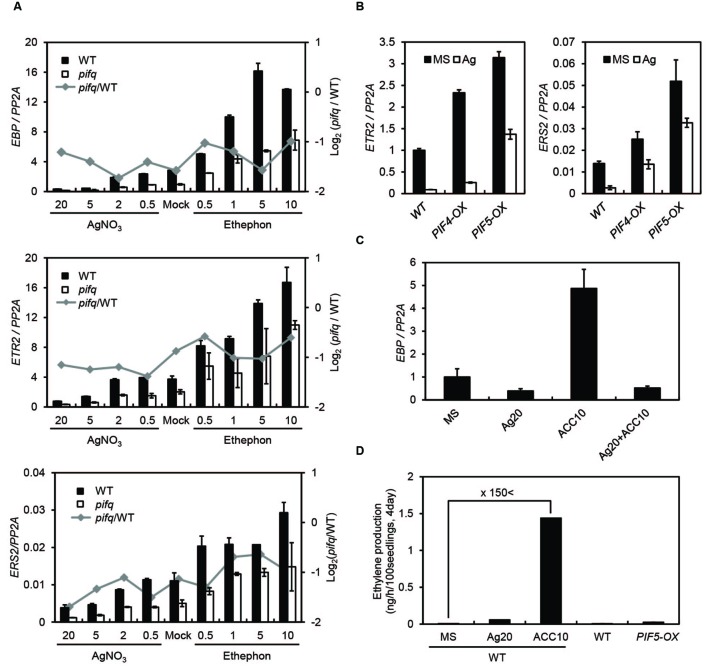
**Phytochrome-interacting factors promote ethylene marker gene expression independent of ethylene. (A)** Expression of the representative ethylene markers *EBP*, *ETR2*, and *ERS2* in 4-day-old WT and *pifq* mutant etiolated seedlings. Seedlings were untreated (mock) or treated with AgNO_3_ or ethephon at the indicated concentrations (μM). The gray line indicates log_2_ fold changes between *pifq* mutants and WT. **(B)** Expression of the representative ethylene markers *ETR2* and *ERS2* in 4-day-old WT, *PIF4-OX*, and *PIF5-OX* etiolated seedlings untreated (mock) or treated with AgNO_3_ (20 μM). **(C)** Expression levels of *EBP* from 4-day-old WT etiolated seedlings untreated (mock) or treated with AgNO_3_ (20 μM), ACC (10 μM), or AgNO_3_+ACC. *PP2A* was used as an internal qPCR control (SD, *n* = 3 biological replicates). **(D)** Quantification of endogenous ethylene from 4-day-old WT and *PIF5-OX* seedlings treated as indicated (SD, *n* = 2 biological replicates).

Since dark-grown *PIF5*-overexpressing seedlings synthesize far more ethylene than wild type seedlings, we asked whether the suppression of ethylene-responsive genes in *pifq* mutants is due to low levels of ethylene production. Thus, we measured endogenous ethylene levels in dark-grown *pifq* mutant seedlings at 24 h intervals (**Figure 1D**). Surprisingly, we found PIFs do not robustly promote ethylene biosynthesis. From 1 to 2 days after germination, we found the *pifq* mutants produce roughly half the ethylene wild type seedlings produce. This pattern reverses after day 2 with the *pifq* mutants producing higher levels of ethylene than wild type (**Figure 1D**). These results are inconsistent with the hypothesis that reduced ethylene biosynthesis is responsible for the suppression of ethylene-responsive genes in *pifq* mutants (**Figure 1C**). We further examined the expression of well-known ethylene-inducible genes (i.e., *EBP*, *ETR2*, and *ERS2*) in the presence of either a saturating level of the ethylene perception inhibitor silver nitrate (AgNO_3_) or the ethylene-producing compound ethephon. Ethylene induces and AgNO_3_ represses these ethylene-inducible markers in both wild type and *pifq* mutants (**Figure 2A**). Interestingly, the expression of these marker genes is lower in the *pifq* mutants than in wild type seedlings at all doses of AgNO_3_ and ethephon (**Figure 2A**). On the other hand, *PIF4*- and *PIF5*-overexpressing lines show increased marker gene expression for the same doses of AgNO_3_ (**Figure 2B**). In these experiments, the AgNO_3_ dose (20 μM) was high enough to fully suppress ethylene responses even with simultaneous treatment of excessive ethylene (**Figures 2C,D**). These results, thus, suggest PIFs promote the expression of ethylene-responsive genes regardless of endogenous ethylene levels.

### PIF Signaling Does Not Significantly Affect Ethylene Signaling Upstream of EIN3

Reduced expression of ethylene signaling genes may explain the reduced responsiveness of ethylene marker genes in *pifq* mutants in response to exogenous ethylene. We thus examined whether PIFs regulate the expression of ethylene signaling genes (Supplementary Figure S2A). Although we did find via microarray (Supplementary Figure S2B) and qRT-PCR (Supplementary Figure S2C) that some ethylene signaling genes are significantly suppressed in *pifq* mutants, the suppressed genes include both positive (EIN2) and negative regulators of ethylene signaling (ETR2 and CTR1). This complicates any prediction of their net effect on ethylene responses. Since ethylene signaling pathway ultimately impinges on EIN3 protein stabilization (Supplementary Figure S2A), we examined whether the ethylene-mediated EIN3 protein stability is affected in the pifq mutant. However, the EIN3 proteins were stabilized by both ACC and ethephon in the pifq mutants, just as it is in wild type (Supplementary Figure S3A). Red light treatment has no effect on ethephon-induced stabilization of EIN3 (Supplementary Figure S3B), nor is *EIN3* expression significantly altered in *pifq* mutants or by red light treatment (Supplementary Figure S3C). These results suggest the transcriptional regulation of ethylene signaling genes by PIFs does not significantly affect ethylene signaling upstream of EIN3. Instead, the altered expression of ethylene responsive genes in *pifq* mutants may relate to the activity of EIN3 itself.

### PIFs and EIN3 Directly Regulate Overlapping Target Genes

**FIGURE 3:**
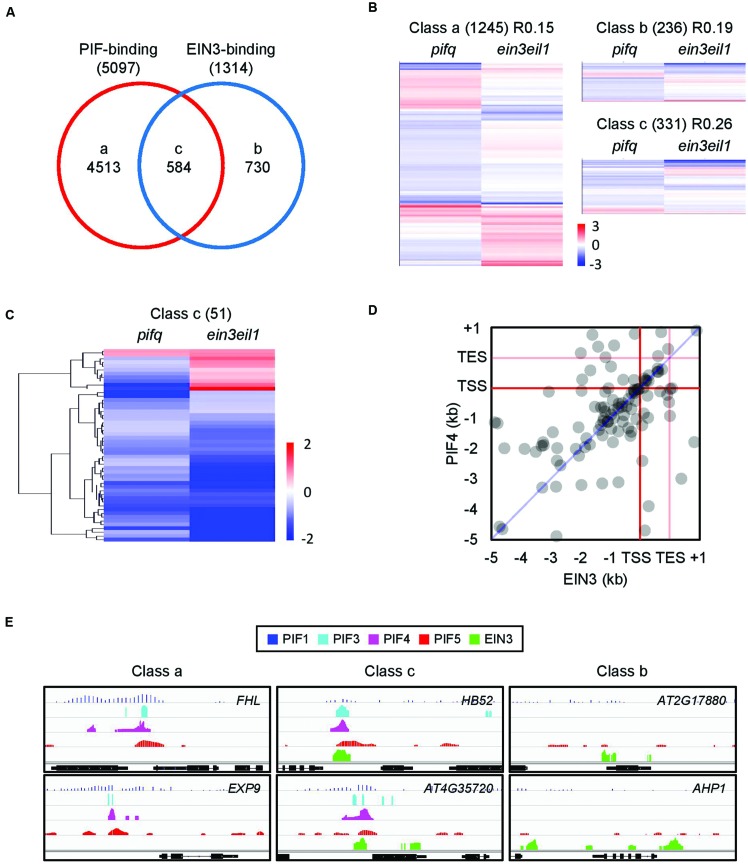
**Phytochrome-interacting factors and EIN3 bind and regulate overlapping target genes. (A)** PIF-binding and EIN3-binding genes significantly overlap. Data were retrieved from relevant ChIP-chip or ChIP-seq databases (Oh et al., 2009, 2012; Hornitschek et al., 2012; Chang et al., 2013; Zhang et al., 2013). Lowercase letters indicate PIF and/or EIN3 binding classes. **(B)** Hierarchical clustering of the expression patterns for each gene class from **(A)**. Gene expression data were retrieved from previous *pifq* and *ein3/eil1* microarrays (Shin et al., 2009; Zhong et al., 2009). Genes whose expression data correspond to *P* < 0.05 in at least one of the two microarrays are shown. **(C)** Hierarchical clustering of the expression patterns for class c genes. Fold changes were rescaled to ± 2 (Log_2_) and only genes whose expression data correspond to *P* < 0.05 in both microarrays are shown. Gene expression data were retrieved from the same source as **(B)**. **(D)** Spatial distribution of PIF4- and EIN3-binding peaks along the promoter (-5 kb to start), coding (TSS, TES) and 3′ (TES to +1 kb) regions of shared target genes. Binding peak data were retrieved from published ChIP-seq experiments performed on PIF4 and EIN3 (Oh et al., 2012; Chang et al., 2013). The analysis was re-done as described in the section “Materials and Methods.”. **(E)** Representative PIF- and EIN3-binding peaks in the promoters of shared or non-shared target genes from the gene classes defined in **(A)**.

It is possible EIN3 activity depends on the activity of the PIFs. By comparing the known genome-wide targets of four PIFs (PIF1, PIF3, PIF4, and PIF5) with those of EIN3, we found a significant number of shared targets (584 genes, hypergeometric test, *p* < 10^-90^; **Figure 3A**). These shared targets account for 11% of the PIF targets and 44% of the EIN3 targets. We next examined *pifq* and *ein3/eil1* microarrays (Shin et al., 2009; Zhong et al., 2009) to determine how PIFs and EIN3 regulate the expression of these shared targets (class c from **Figure 3A**). Although only 331 of the 584 shared targets were included in the microarray analyses (**Figure 3B**), we found 51 of 331 are significantly regulated by both PIFs and EIN3/EIL1, mostly in the same direction (**Figure 3C**). We observed enriched binding of both PIF4 and EIN3 in the promoter regions of their shared targets (Supplementary Figure S4B). Furthermore, many of the PIF4 and EIN3 binding peaks precisely overlap (**Figures 3D,E**) and most of the binding peaks fall within 200 bp of each other (Supplementary Figure S4C, blue). In other words, these two transcription factors bind closely to one another on their shared target promoters. To exclude the possibility that this proximity of the PIF4 and EIN3 binding peaks is attributable to chance, we selected PIF4 and EIN3 binding peaks from random target promoters instead of from the same promoter and then calculated the distances between each peak (Supplementary Figure S4A). Compared to the random peak distances (Supplementary Figure S4C, orange), we found the actual PIF4-EIN3 peaks (Supplementary Figure S4C, blue) are strongly biased toward shorter inter-peak distances. This supports the binding of PIFs and EIN3 to their shared targets in close proximity.

### PIFs and EIN3 Bind Independently to Their Target Promoters

**FIGURE 4:**
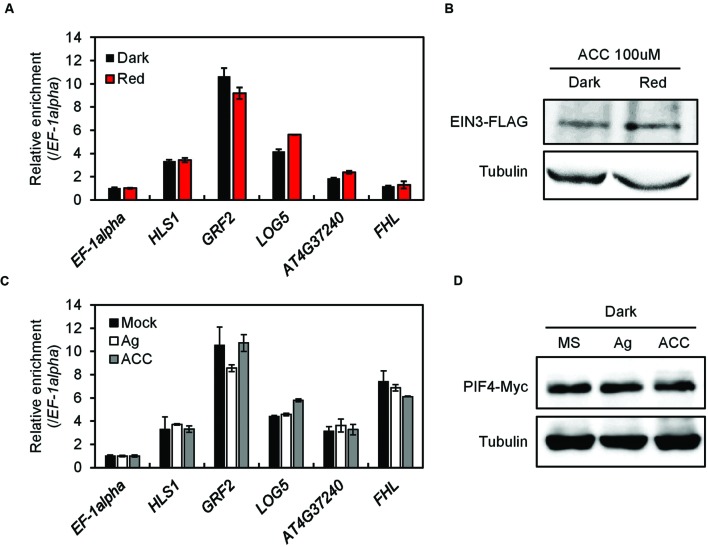
**Phytochrome-interacting factors and EIN3 bind independently to their shared target promoters. (A,C)** ChIP assay for EIN3 and PIF4 binding to shared target promoters (*HLS1*, *GRF2*, *LOG5*, and *AT4G37240*) under dark and red light conditions **(A)** or under darkness untreated or treated with 20 μM AgNO_3_ or 10 μM ACC **(C)**. Fold enrichment was calculated by normalizing the results with respect to an input control and a control region, *EF-1-alpha* (SD, *n* = 3 biological replicates). *FHL* was used as a PIF-specific target gene. **(B,D)** EIN3-FLAG and PIF4-Myc protein levels were measured under the indicated conditions. Tubulin was used as a loading control.

The proximity of the PIF and EIN3 binding peaks suggests PIFs and EIN3 may enhance one another’s binding to their shared target promoters. Since red light dissociates PIFs from their target promoters and enhances PIF degradation (Park et al., 2004, 2012; Shen et al., 2005; Al-Sady et al., 2006; Oh et al., 2006; Lorrain et al., 2008), it may also inhibit EIN3 binding to promoters it co-targets with PIFs. We therefore performed a ChIP assay with transgenic plants expressing FLAG-tagged EIN3 grown either in the dark or under red light (**Figure 4A**). After confirming EIN3 protein stability is unaffected by red light (**Figure 4B**), we found red light does not significantly affect EIN3 binding to four PIF co-targeted promoters (i.e., those of *HLS1*, *GRF2*, *LOG5*, and *SOB3*) or to two non-binding control promoters (i.e., those of *EF-1alpha* and *FHL*; **Figure 4A**). This suggests EIN3 binding to the target promoters it shares with the PIFs is independent of PIF binding. We next asked whether ethylene signaling enhances PIF binding to co-targeted promoters by performing a ChIP assay with transgenic plants expressing MYC-tagged PIF4 grown in the presence of either AgNO_3_ or ACC (**Figure 4C**). Since ethylene stabilizes EIN3, AgNO_3_ should inhibit and ACC should enhance the binding of PIF4 to PIF4/EIN3 co-targeted promoters if EIN3 is required for PIF4 binding. After confirming neither AgNO_3_ nor ACC treatment significantly alters PIF4 protein stability (**Figure 4D**), we found PIF4 binds equally to four co-targeted promoters regardless of the presence of AgNO_3_ or ACC (**Figure 4C**). This suggests the binding of PIF4 to its target promoters is independent of ethylene signaling. Together, these results suggest PIFs and EIN3 independently bind their target promoters.

### PIFs and EIN3 Regulate the Expression of Their Shared Targets Either Interdependently or Additively

**FIGURE 5:**
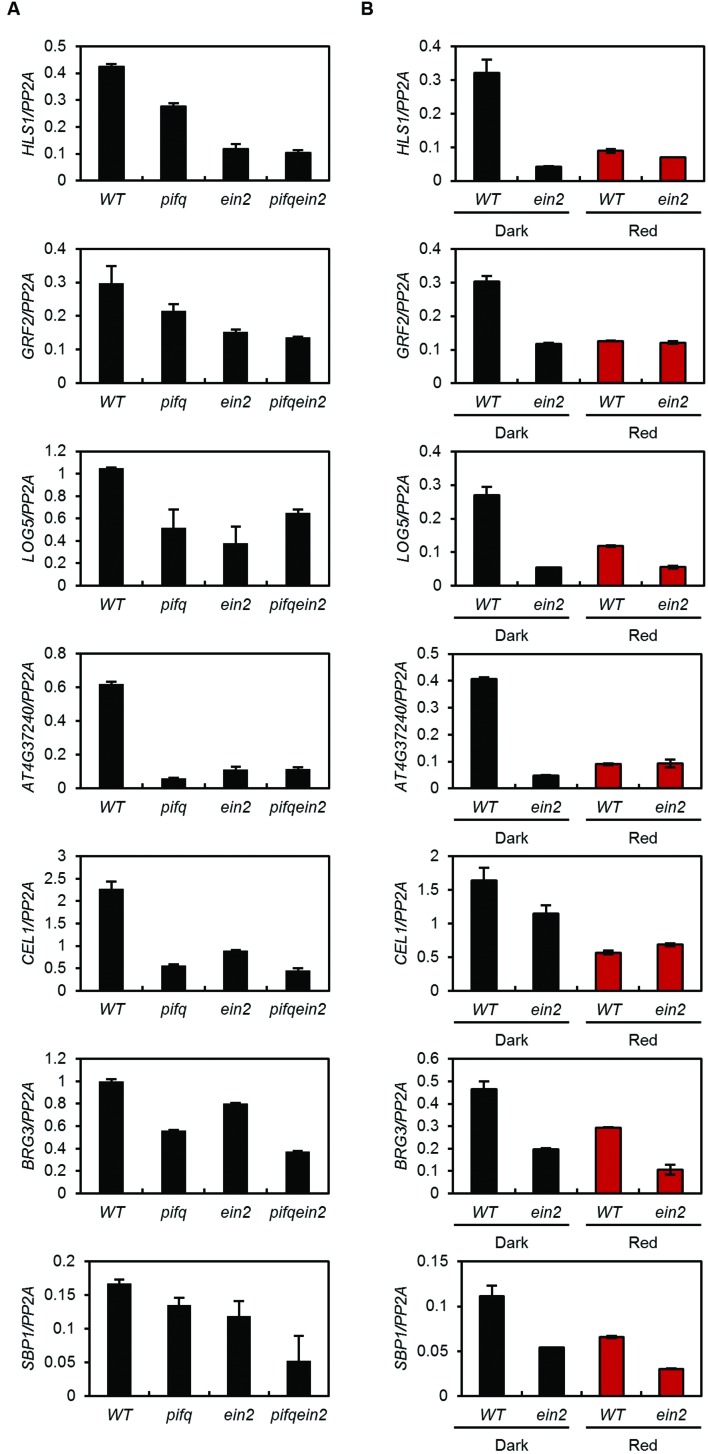
**Phytochrome-interacting factors and EIN3 interdependently activate a subset of their shared target genes**. Expression of shared target genes in 4-day-old WT, *pifq*, *ein2*, and *pifq ein2* etiolated seedlings treated with 250 nM ACC **(A)** or in 4-day-old WT and *ein2* MS-medium-grown seedlings under dark or red light conditions **(B)**. *PP2A* was used as an internal control (SD, *n* = 3 biological replicates).

Phytochrome-interacting factors and EIN3 bound to the same promoters may independently or interdependently regulate the expression of their shared targets. We therefore measured the expression of their shared targets in *pifq*, *ein2*, and *pifq;ein2* quintuple mutants. EIN3 and EIL protein levels are very low in *ein2* mutants because they are constitutively degraded by EBF1 and EBF2 (Guo and Ecker, 2003; Potuschak et al., 2003). Some of the shared target genes (e.g., *LOG5* and *AT4G37240*) are equally repressed in the *pifq*, *ein2*, and *pifq;ein2* mutants compared to wild type, suggesting PIFs and EIN3 interdependently activate their expression (**Figure 5A**). The expression levels of other shared target genes (e.g., *HLS1* and *GRF2*) are slightly higher in the *pifq* mutant than in the *ein2* and *pifq;ein2* mutants (**Figure 5A**). Since the rest of the PIFs (e.g., PIF7) remain active in *pifq* mutants, they may be responsible for the residual *HLS1* and *GRF2* expression observed in *pifq* mutants. To remove the residual PIF activities, we treated wild type seedlings with red light because red light robustly suppresses all PIFs (including PIF7) via Pfr phytochrome (Leivar et al., 2008a). The red light treatment of wild type seedlings reduced shared target expression to the levels observed in *ein2* mutants and unlike in wild type seedlings, *ein2* mutants showed low expression of co-targeted genes regardless of red light treatment (**Figure 5B**). On the other hand, the expression of shared target genes (*HLS1* and *LOG5*) in *ein3;eil1* double mutants was reduced by the red light treatment (Supplementary Figure S5) suggesting that the activity of residual EIN3-like protein (e.g., EIL2) is also dependent on PIF activities. Taken together, our results suggest both PIFs and ethylene signaling are required for high expression of these co-targeted genes in the dark. However, not all the shared target genes are interdependently regulated by PIFs and EIN3 as shown by further decreased expression of other subset of co-targeted genes (*BRG3* and *SBP1*; **Figure 5B**). These results support the hypothesis that PIFs and EIN3 either interdependently or additively activate the expression of their shared targets.

### HLS1 Regulates the Expression of Chlorophyll Biosynthesis Genes

**FIGURE 6:**
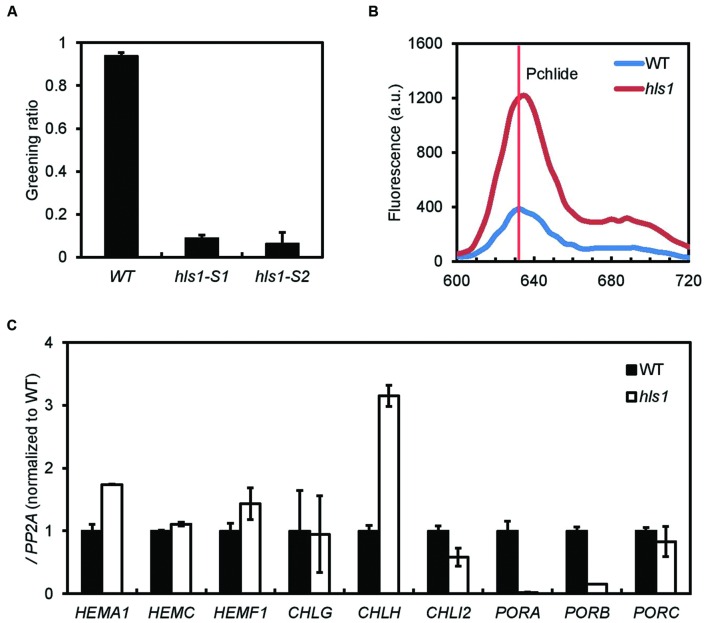
***Hookless1* inhibits photobleaching by regulating chlorophyll synthesis gene expression. (A)** Excessive photobleaching of *hls1* mutants during the dark to light transition (SD, *n* = 2 independent experiments; *n* > 40 for each experiment). **(B)** Increased protochlorophyllide levels in dark-grown *hls1* mutants as measured by fluorescence. **(C)** Expression levels of chlorophyll synthesis genes in 4-day-old WT and *hls1* etiolated seedlings. *PP2A* was used as an internal control (SD, *n* = 3 biological replicates).

*Hookless1* mediates the ethylene-induced formation of the apical hook. Since HLS1 is one of the genes co-targeted by PIFs and EIN3 and since PIFs and EIN3 are required for the prevention of photobleaching in etiolated seedlings, we asked whether HLS1 is also involved in the prevention of photobleaching. When 4-day-old etiolated wild type seedlings are transferred to white light, their cotyledons turn green. However, the cotyledons of *hls1* mutant seedlings fail to turn green and are instead photobleached (**Figure 6A**). Photobleaching occurs when protochlorophyllide over-accumulates, and etiolated *hls1* seedlings consistently accumulate more protochlorophyllide than wild type seedlings (**Figure 6B**). We therefore measured the expression of a series of chlorophyll biosynthesis genes in the *hls1* mutants. Consistent with their photobleaching phenotype, *hls1* mutant seedlings show high expression of *HEMA1* and *CHLH* and low expression of *PORA* and *PORB*. Together, these results suggest PIFs and EIN3 prevent photo-oxidative damages of etiolated seedlings in the dark to light transition by activating *HLS1*, which represses *HEMA1* and *CHLH* and activates *PORA* and *PORB*.

## Discussion

**FIGURE 7:**
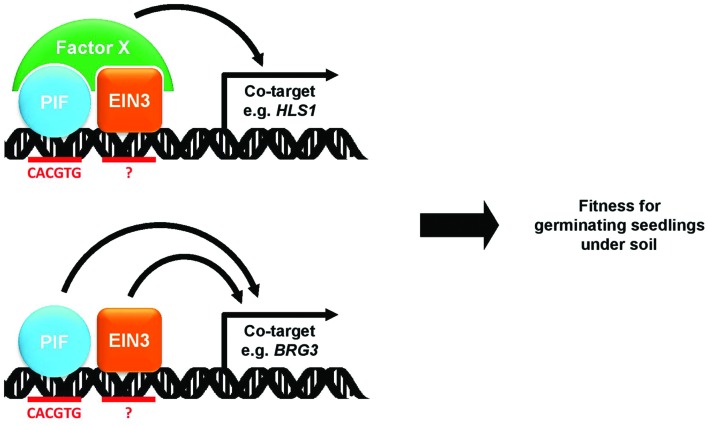
**A model for the interdependent and additive activation of shared PIF and EIN3 target genes**. PIF and EIN3 transcription factor bind to shared targets in a close proximity. In the interdependent mode (upper), two transcription factors recruit factor X which in turn activates the target gene expression. In the additive mode (bottom), two transcription factors independently activate the target gene expression. Through both modes of target gene regulation, PIF and EIN3 increase fitness of seedlings that germinate under soil.

The phytochrome and ethylene signaling pathways antagonistically regulate apical hook formation and chlorophyll biosynthesis in etiolated seedlings. The precise molecular integrations between these two signaling pathways in seedling development, however, are unknown. In this study, we present evidence the phytochrome and ethylene signaling pathways are integrated at the level of the transcriptional control of shared targets by the PIFs and EIN3. We found via microarray analysis a down-regulation of ethylene-responsive genes in *pifq* mutants. Since the *pifq* mutants show neither reduced ethylene nor reduced EIN3 protein levels, the down-regulated ethylene response suggests a reduction in EIN3 activity in the absence of PIFs. We found via ChIP analysis that PIFs and EIN3 share a significant number of direct target genes, which they either interdependently or additively activate (**Figure 7**). HLS1, one of these shared PIF/EIN3 targets, is required in etiolated seedlings to prevent photobleaching and to form the apical hook. This demonstrates the phytochrome and ethylene signaling pathways converge at the promoters of genes co-regulated by PIFs and EIN3.

In a microarray analysis, we found a significant overlap between PIF- and ethylene-regulated genes in etiolated seedlings. Consistent with the similar roles these signaling pathways play in etiolated seedling development, most of these overlapping target genes are regulated by PIFs and EIN3/EIL1 in the same direction (**Figure 3C**). According to published reports, PIFs activate the expression of some ethylene biosynthesis genes including *ACSs*, and *PIF5-OX* seedlings produce more ethylene than wild type seedlings (Khanna et al., 2007). We found, however, *pifq* mutants only produce less ethylene than wild type for the first 2 days post-germination. After that, *pifq* mutants produce more ethylene than wild type (**Figure 1D**). Thus, PIF-induced ethylene biosynthesis cannot be responsible for the reduced expression of ethylene-responsive genes in 4-day-old *pifq* mutant seedlings (**Figure 1C**). Moreover, *PIF4/5-OX* increases the expression of ethylene-responsive genes even in the presence of saturating concentrations of the ethylene perception inhibitor AgNO_3_ (**Figure 2B**). These results suggest PIFs directly regulate ethylene signaling independent of ethylene biosynthesis and ethylene perception.

The *pifq* mutants also show repression of some ethylene signaling components compared to wild type. These repressed genes, however, include both positive and negative regulators of ethylene responses (Supplementary Figure S2). In addition, neither the *pifq* mutation nor red light treatment alter EIN3 protein levels (Supplementary Figures S3A,B; **Figure 4B**). Since EIN3 protein levels are tightly regulated by ethylene signaling (Guo and Ecker, 2003; Potuschak et al., 2003), PIFs likely affect ethylene signaling downstream rather than upstream of EIN3. Indeed, we found PIFs cooperate with EIN3 to regulate the expression of ethylene-responsive genes. Our ChIP-Seq analyses showed PIFs and EIN3 share many target genes and bind to their co-regulated promoters in close proximity (**Figures 3A,D,E**). This suggests the large overlap we observed between PIF- and ethylene-regulated genes is due to extensive overlap of the DNA binding loci of the PIFs and EIN3. Furthermore, PIFs and EIN3 interdependently activate the expression of a subset of their shared targets (**Figure 5**). This ensures the transcriptional activation of these shared target genes only when light signaling is inactive and ethylene signaling is active.

We expected these two transcription factors interdependently regulate gene expression by enhancing one another’s DNA-binding ability, presumably via a direct protein–protein interaction. We were unable to observe, however, any change in PIF4 binding to PIF/EIN3 shared target promoters in response to ethylene or AgNO_3_ treatment (**Figure 4C**). We were also unable to observe any change in EIN3 binding in response to red light treatment (**Figure 4A**) even though red light dramatically reduces PIF levels. It thus seems the PIFs and EIN3 bind their shared targets independent of one another. It is also possible, though, PIFs and EIN3 cooperatively recruit transcriptional co-activators or chromatin modifying enzymes to activate target gene expression (**Figure 7**). PIF3 is known to interact with the chromatin remodeling factor PICKLE to regulate gene expression, and PICKLE is required for hypocotyl elongation and apical hook formation in seedling etiolation (Zhang et al., 2014). In a future study, we will determine whether PICKLE is recruited cooperatively by PIFs and EIN3 to their shared target promoters.

Both PIFs and EIN3 protect etiolated seedlings from photo-oxidative damage upon sudden exposure to light, enhancing survival. Several mechanisms have been proposed to account for this protective effect. PIF1 and PIF3 inhibit the accumulation of protochlorophyllide in the dark by repressing chlorophyll biosynthesis genes like *HEMA1* and *CHLH* (Shin et al., 2009; Stephenson et al., 2009). In addition, PIF1 directly activates *PORC* expression and indirectly activates *PORA* and *PORB* expression (Moon et al., 2008). Ethylene-activated EIN3 directly binds the promoters of *PORA* and *PORB* to activate their expression (Zhong et al., 2009). EIN3 also directly increases *PIF3* expression (Zhong et al., 2012), which, in turn, inhibits the accumulation of protochlorophyllide (Zhong et al., 2014). In addition to these mechanisms, we propose PIFs and EIN3 indirectly regulate chlorophyll biosynthesis through HLS1, which is known to regulate ethylene-induced apical hook formation (Lehman et al., 1996). We found PIFs and EIN3 cooperatively regulate *HLS1* expression (**Figures 4A,C** and 5; Supplementary Figure S4), and HLS1 is required for preventing photo-oxidative damage (**Figures 6A,B**). HLS1 also represses *HEMA1* and *CHLH* expression and activates *PORA* and *PORB* expression (**Figure 6C**). Thus, PIFs and EIN3 directly and indirectly regulate chlorophyll biosynthesis gene expression via their shared target HLS1. This ensures etiolated seedlings complete the greening process without photo-oxidative damage when they are exposed to light.

Phytochrome signaling is interconnected with various hormone signaling pathways. PIF4 directly interacts with the BR-regulated transcription factor BZR1 and the auxin-regulated transcription factor ARF6 (Oh et al., 2012, 2014). The interactions interdependently regulate the expression of thousands of target genes to achieve proper hypocotyl elongation by integrating phytochrome, brassinosteroid, and auxin signalings. Our study demonstrates a similar integration of phytochrome signaling with the ethylene signaling pathway via the transcriptional co-regulation of targets shared by PIFs and EIN3. The co-regulation of shared targets by key signaling transcription factors seems to be a common mechanism integrating phytochrome signaling with hormonal signaling. Since PIF4 directly interacts with other hormone signaling transcription factors, it is possible that PIFs directly interact with EIN3 to co-regulate target genes, which should be determined by a future study.

## Author Contributions

JJ, EO, and GC designed the study. JJ, KK, and EO performed the overall experiments. JJ performed bioinformatics analysis. MK and GH consulted and performed the gas chromatography. HK and OP consulted and performed the Western blots of native EIN3 protein. Y-IP and GC supervised the work. JJ, EO, and GC wrote the manuscript. All authors discussed the results and made substantial contributions to the manuscript.

## Conflict of Interest Statement

The authors declare that the research was conducted in the absence of any commercial or financial relationships that could be construed as a potential conflict of interest. The reviewer HQ and handling Editor declared their shared affiliation, and the handling Editor states that the process nevertheless met the standards of a fair and objective review.
