# Supplementary figures and images for: Phytochrome and Ethylene Signaling Integration in Arabidopsis Occurs via the Transcriptional Regulation of Genes Co-targeted by PIFs and EIN3
Source: Front Plant Sci. 2016 Jul 19;7:1055. doi: 10.3389/fpls.2016.01055 (PMC4949226; doi:10.3389/fpls.2016.01055)

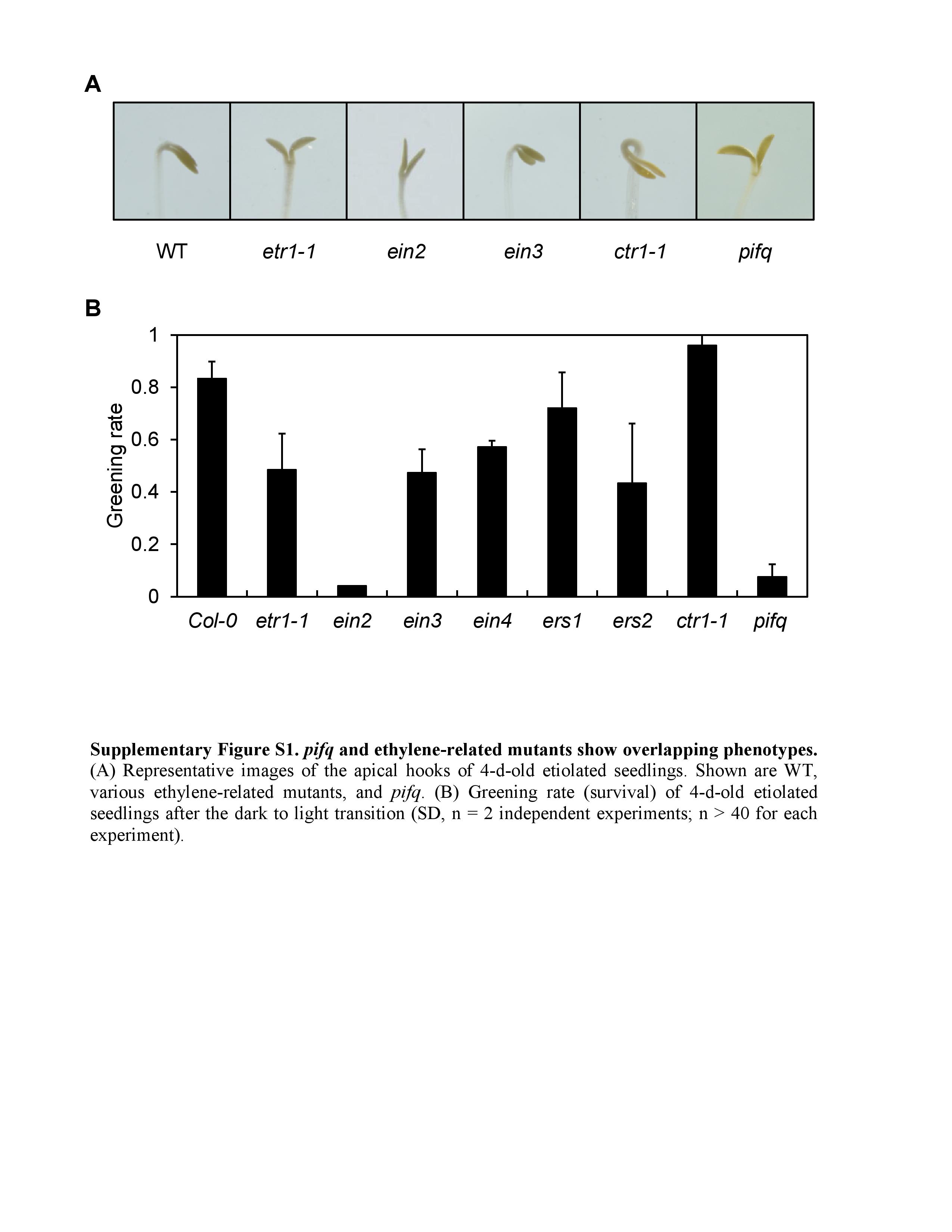

Supplement: Supplementary file 3 [file Image_1.JPEG]

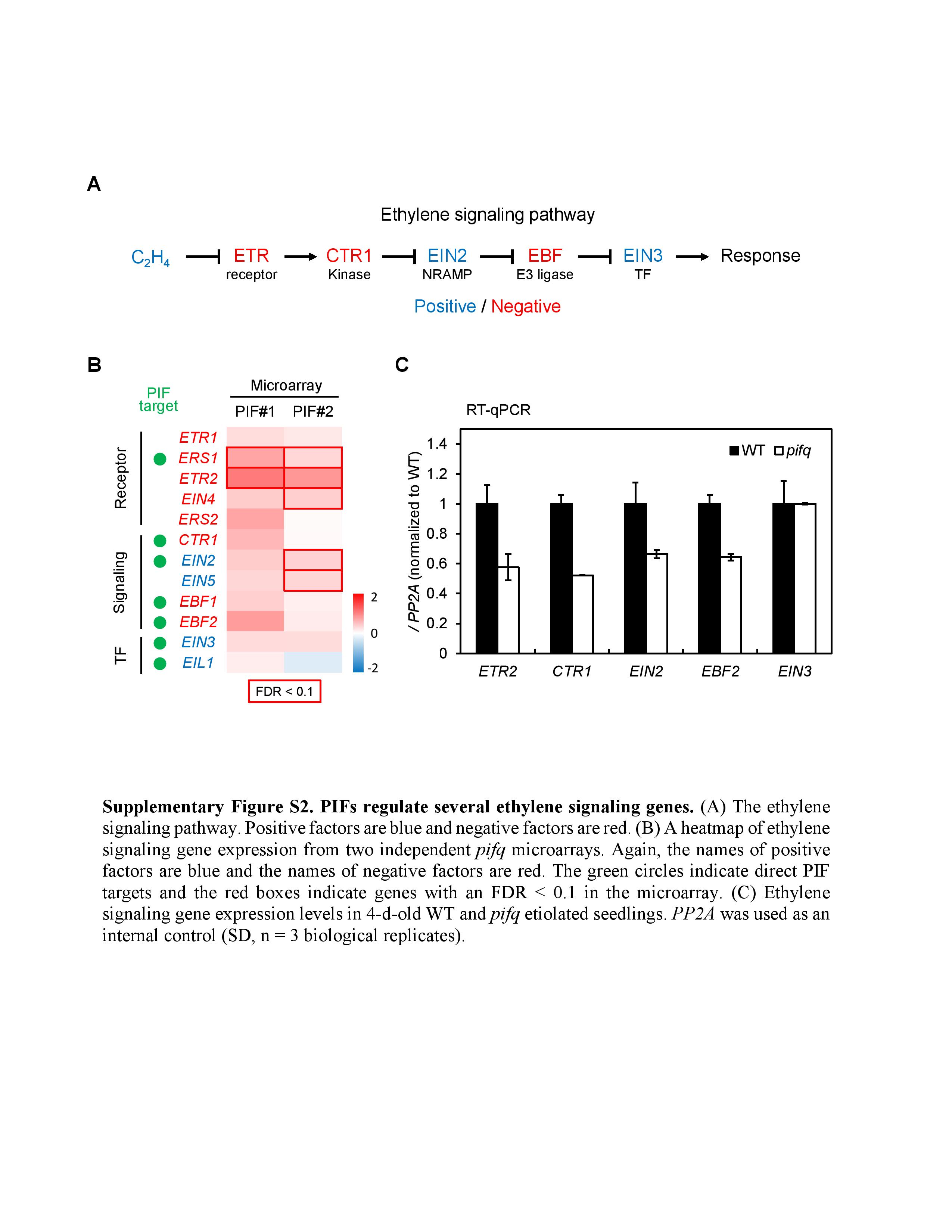

Supplement: Supplementary file 4 [file Image_2.JPEG]

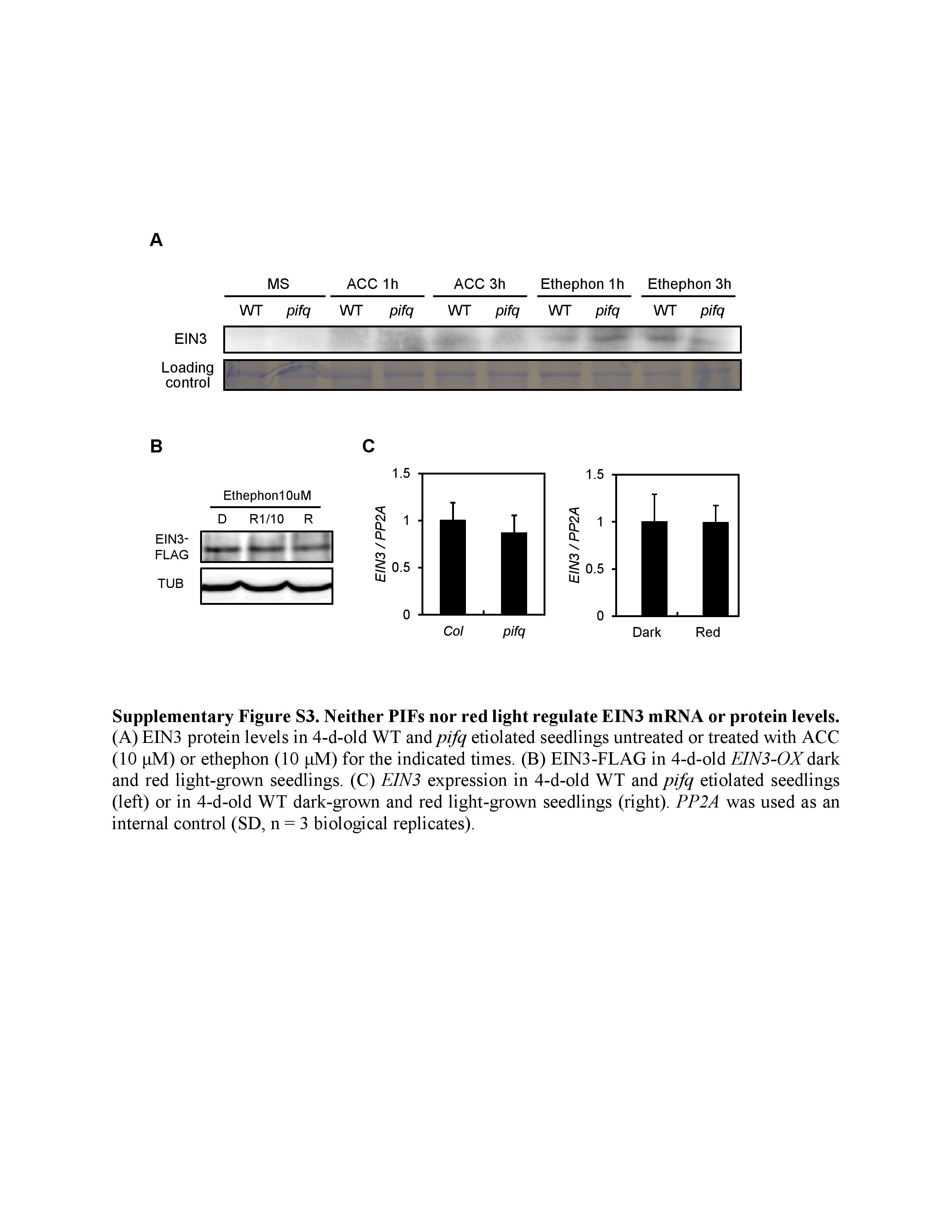

Supplement: Supplementary file 5 [file Image_3.JPEG]

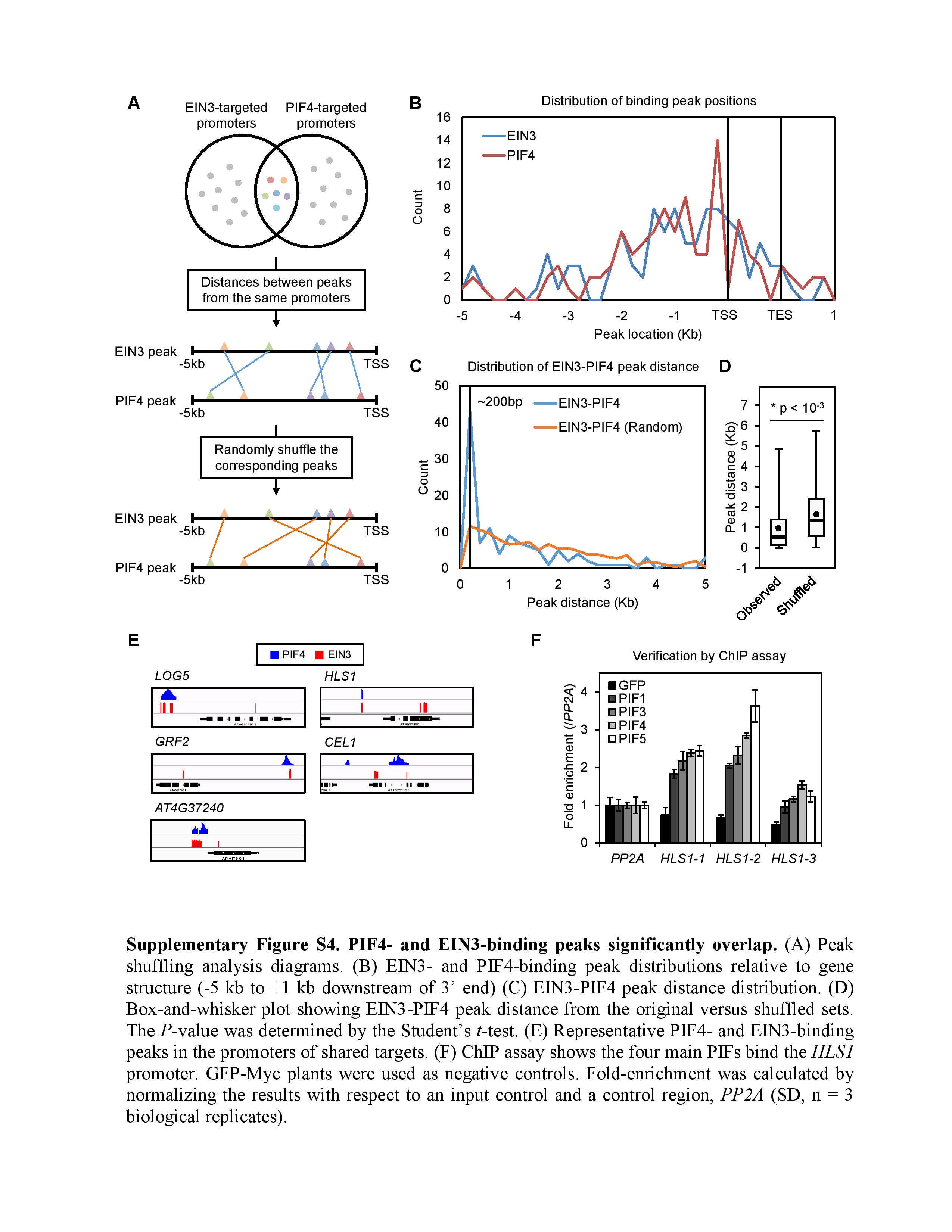

Supplement: Supplementary file 6 [file Image_4.JPEG]

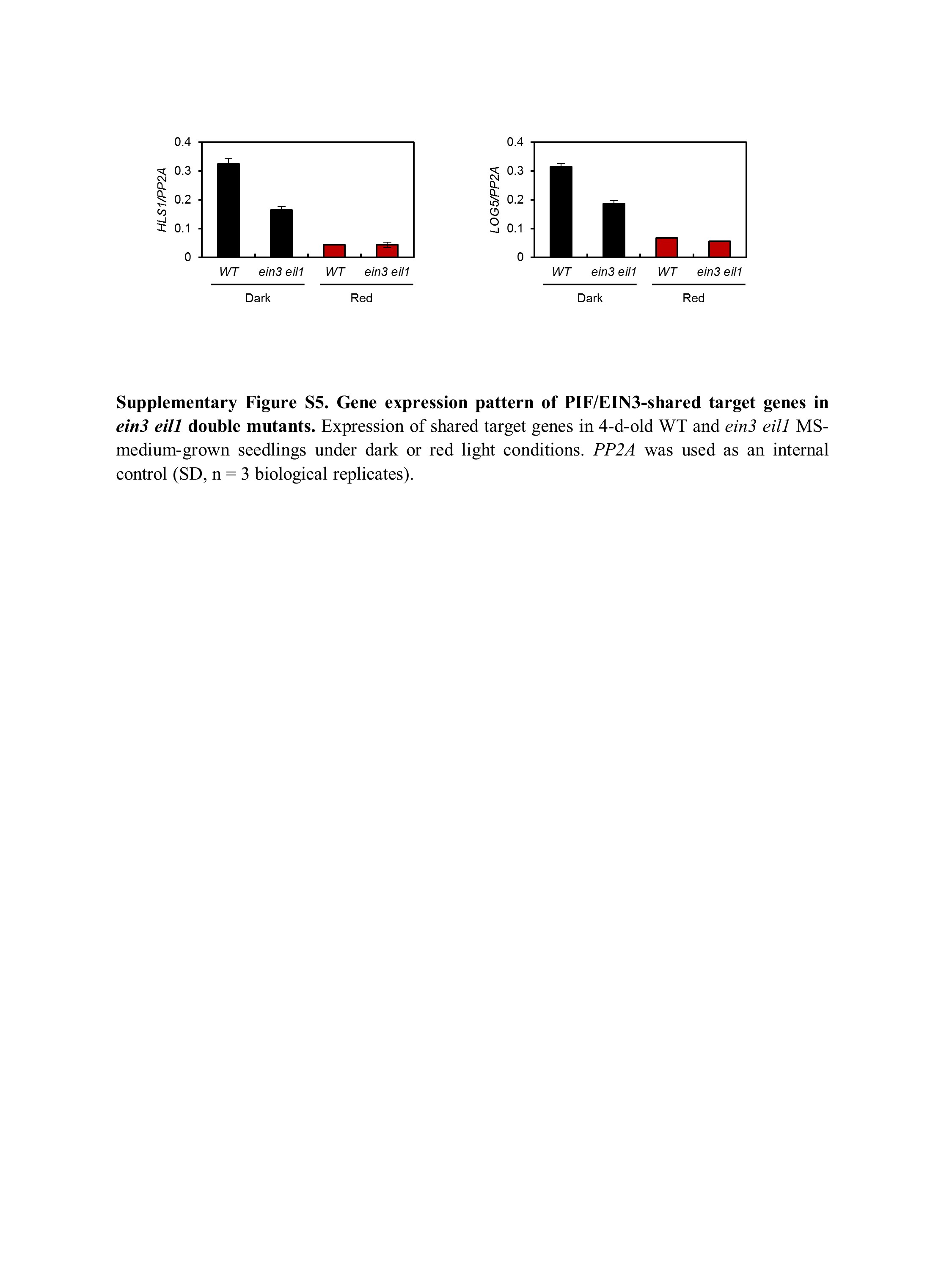

Supplement: Supplementary file 7 [file Image_5.JPEG]
